# Chitosan-Based Flexible Memristors with Embedded Carbon Nanotubes for Neuromorphic Electronics

**DOI:** 10.3390/mi12101259

**Published:** 2021-10-17

**Authors:** Jin-Gi Min, Won-Ju Cho

**Affiliations:** Department of Electronic Materials Engineering, Kwangwoon University, Gwangun-ro 20, Nowon-gu, Seoul 01897, Korea; wlsrl1659@naver.com

**Keywords:** chitosan, single-walled carbon nanotube random network, memristor, analog switching, neuromorphic, synaptic weight change

## Abstract

In this study, we propose high-performance chitosan-based flexible memristors with embedded single-walled carbon nanotubes (SWCNTs) for neuromorphic electronics. These flexible transparent memristors were applied to a polyethylene naphthalate (PEN) substrate using low-temperature solution processing. The chitosan-based flexible memristors have a bipolar resistive switching (BRS) behavior due to the cation-based electrochemical reaction between a polymeric chitosan electrolyte and mobile ions. The effect of SWCNT addition on the BRS characteristics was analyzed. It was observed that the embedded SWCNTs absorb more metal ions and trigger the conductive filament in the chitosan electrolyte, resulting in a more stable and wider BRS window compared to the device with no SWCNTs. The memory window of the chitosan nanocomposite memristors with SWCNTs was 14.98, which was approximately double that of devices without SWCNTs (6.39). Furthermore, the proposed SWCNT-embedded chitosan-based memristors had memristive properties, such as short-term and long-term plasticity via paired-pulse facilitation and spike-timing-dependent plasticity, respectively. In addition, the conductivity modulation was evaluated with 300 synaptic pulses. These findings suggest that memristors featuring SWCNT-embedded chitosan are a promising building block for future artificial synaptic electronics applications.

## 1. Introduction

Owing to the von Neumann bottleneck, conventional computing systems face enormous challenges when dealing with real-time decision-making processes and large amounts of unstructured data and processing large amounts of information [1]. Therefore, efficient, high-performance computing systems are considered the new benchmarks for rapid processing of big data [2,3]. Regarding the implementation of such computing systems, two-terminal memristors with metal–insulator–metal structures have been studied extensively owing to their geometric simplicity, nonvolatile memory, low operating power consumption, and ability to perform computations based on successive analog resistive switching (RS) in the insulating layer [4,5,6]. Various materials, including bio-inspired, organic, inorganic, and hybrid nanocomposites, have been explored for the RS layer of memristors. Among them, bio-inspired organic materials, such as chitosan, cellulose, albumen, and gelatin, are attracting considerable attention [7]. Prospective materials must be suitable for integration with advanced electronics, such as skin-attachable and wearable devices, which are fabricated on transparent, flexible, and stretchable substrates as opposed to rigid substrates [8,9]. Accordingly, solution-based, low-temperature processable natural organic materials offer a variety of processing options and are a viable alternative to inorganic-based solutions because of their good biodegradability and bio-absorbability and non-toxicity [7,10]. Moreover, bio-organic memristors should overcome challenges such as low endurance and unstable long-term retention. Among bio-inspired organic materials, several advantages identify chitosan electrolyte as a particularly suitable candidate for the RS layer of memristors: (1) although chitosan is naturally insulating, adding an acidic solution can increase its ionic conductivity, (2) its amine and hydroxyl groups react readily with metal ions, (3) after cellulose, chitin, the primary component of chitosan, is the most naturally abundant polysaccharide, (4) chitosan is a benign and biodegradable polymer, and (5) chitosan flakes and powder dissolve in diluted acetic acid solutions [11,12,13,14].

In addition, carbon nanotubes (CNTs) are widely applicable carbon-based nanomaterials with extraordinary physicochemical properties, such as low-temperature processability, transparency, elasticity, and high mobility [15,16,17,18]. Significantly, drop-casting-based solution processes enable the formation of highly uniform CNT random networks and, because of their high aspect ratio, strong mechanical strength, and high modulus, CNTs represent useful polymer supplements [19,20]. Therefore, CNTs are used in a variety of applications, including solar cells, supercapacitors, and electric double-layer capacitors (EDLCs) [21,22]. Furthermore, the high thermal conductivity of CNTs makes them suitable as a nanofiller to increase the thermal conductivity of polymeric materials, and extensive studies have been conducted regarding the high internal resonance and large energy exchange properties of polymer/CNT nanocomposite systems [23,24,25,26].

Inspired by the properties of chitosan and CNTs, we fabricated a two-terminal memristor comprising a single-walled CNT (SWCNT) random network embedded in a chitosan film as a functional RS layer on a flexible, transparent polyethylene naphthalate (PEN) substrate. To verify the efficiency of the SWCNT random network in the proposed memristor, an RS layer without SWCNTs was prepared for comparison. Specifically, the RS operation, endurance, retention, and analog memristive switching characteristics were evaluated. Furthermore, the short- and long-term plasticity, which are important for synaptic computation and information storage, were analyzed.

## 2. Experimental

### 2.1. Materials

The following materials were used to fabricate the two-terminal memristors: PEN substrates (125 μm thick; AMG Co., Seoul, Korea), glass substrates (7059 glass; Corning Inc., New York, NY, USA), cool-off-type adhesive (Intelimer^TM^ Tape CS2325NA4; Nitta Corp., Tokyo, Japan), Ti pellets (purity > 99.999%; TFN, Seoul, Korea), Pt pellets (purity > 99.95%; TFN, Korea), poly-L-lysine solution (0.1% (*w*/*v*) in H_2_O; Sigma Aldrich, Saint Louis, MO, USA), SWCNT solution (IsoNanotubes-S: average diameter = 1.4 nm, average length = 300 nm; Nanointegris, Quebec, Canada), chitosan powder (derived from shrimp shell, medium molecular weight 190–310 kDa, deacetylation degree > 75%; Sigma-Aldrich, Seoul, Korea), and acetic acid solution (purity > 99%; Sigma-Aldrich).

### 2.2. Chitosan Solution Manufacturing Process

A bio-compatible chitosan solution was synthesized by dissolving chitosan powder in acetic acid solution. First, the chitosan powder extracted from medium-molecular-weight shrimp shells was dissolved in an acetic acid solution diluted with deionized (DI) water, which was stirred continuously at 800 rpm at 50 °C for 6 h using a magnetic stirrer. Subsequently, the mixture was filtered using a polytetrafluoroethylene syringe filter with a 5 μm pore size to remove impurities. Finally, the filtered solution was cooled at room temperature for 12 h.

### 2.3. Fabrication of Flexible Transparent Memristors

**Figure 1:**
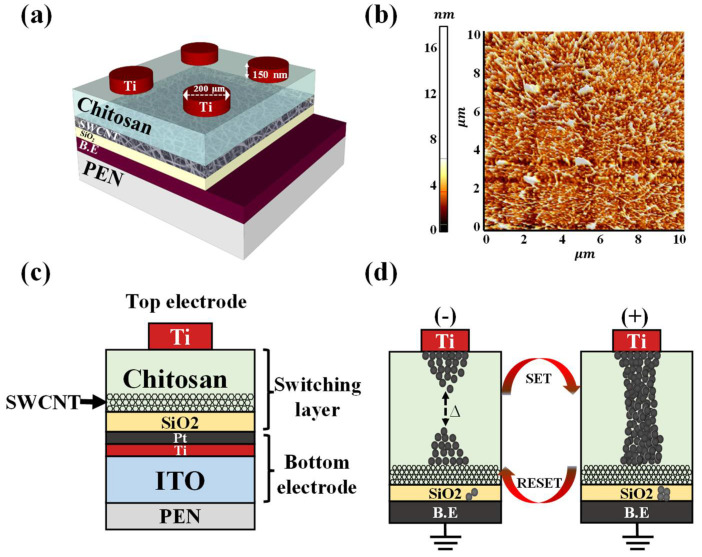
Overview of the developed memristors. (**a**) Schematic diagram of chitosan–SWCNT nanocomposite memristors. (**b**) atomic force microscopy (AFM) image of the single-walled carbon nanotube (SWCNT) random network (10 µm × 10 µm). (**c**) Cross-sectional view of the chitosan–SWCNT nanocomposite memristors. (**d**) Simplified mechanism of filament formation and rupture.

First, PEN substrates were attached to the rigid glass substrates using cool-off-type adhesive to prevent the PEN substrates from shrinking and expanding during the fabrication process. At the end of the process, the flexible, transparent PEN substrates were separated from the glass substrates without incurring physical damage. To form the bottom electrode (BE), a 100 nm thick indium tin oxide (ITO) film was deposited on the PEN substrates using a radio-frequency (RF) magnetron sputtering system at a working pressure of 3.0 mTorr, RF power of 100 W, and an Ar gas flow rate of 20 sccm. Then, Ti and Pt layers (both 10 nm thick) were deposited sequentially using an e-beam evaporator deposition system. The process of embedding the SWCNT random network in the chitosan layers, which is crucial for stable RS, was conducted as follows: First, a 20 nm thick SiO_2_ layer was deposited by RF magnetron sputtering at a working pressure of 4.0 mTorr, RF power of 200 W, and an Ar/O_2_ mixed gas flow rate of 30/2 sccm. Next, a poly-L-lysine solution was drop-cast onto the SiO_2_ thin film surface at room temperature for 1 h, resulting in the formation of an amine-termination layer that serves as an effective SWCNT adhesive layer [27]. Then, the surface of the amine-functionalized SiO_2_ layer was washed with DI water to remove any excess poly-L-lysine solution and dried under a flow of N_2_ gas. Then, the SWCNT solution was drop-cast onto the amine-functionalized SiO_2_ layer to form the SWCNT random network, with the sample maintained at room temperature for 30 min. Then, any excess SWCNT solution was removed by washing with DI water and the sample was blow-dried with N_2_ gas. Next, the chitosan electrolyte solution was spin-coated onto the SWCNT random network at 6000 rpm for 30 s, dried in ambient air for 24 h, and hot-plate oven-baked at 80 °C for 10 min using 2 wt% of chitosan powder. The thickness of the baked chitosan layer was 80 nm. Finally, a 150 nm thick Ti top electrode (TE) with a diameter of 200 µm was deposited onto the RS layer using an e-beam evaporator deposition system and a shadow mask. To determine the effect of the SWCNT random network, comparative memristors without SWCNT random networks were fabricated. Figure 1a,b shows a schematic diagram and an atomic force microscopy (AFM) image of the SWCNT random network (10 µm × 10 µm). It was confirmed that the SWCNT random network was uniformly formed through the drop-casting method. Figure 1c,d shows a cross-sectional view of the chitosan–SWCNT memristors and a simplified mechanism of filament formation and rupture.

### 2.4. Characterization Methods

**Figure 2:**
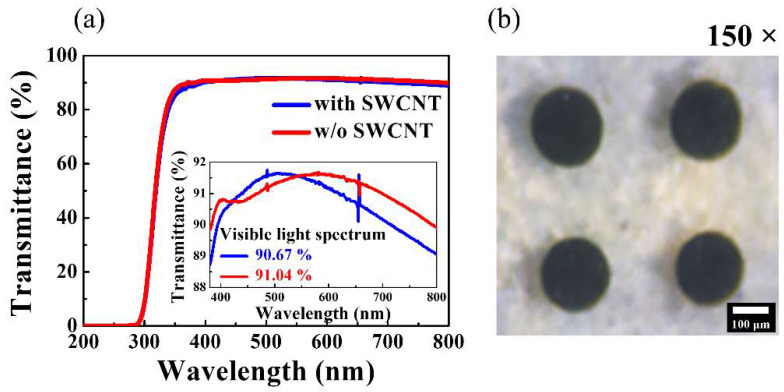
(**a**) Optical transmittance spectra of chitosan nanocomposite memristors with and without SWCNTs. The inset shows the spectra between transmittance values of 88% and 92% for visible wavelengths (380–800 nm). (**b**) Optical microscopy image of chitosan–SWCNT nanocomposite memristors.

The fabricated flexible, transparent memristors were placed in a dark box to protect them from external electrical and light noise. The RS operation and memristive synaptic behavior were measured using an Agilent 4156B precision semiconductor parameter analyzer (Hewlett-Packard Co., Palo Alto, CA, USA). To measure the synaptic behavior, electrical pulse stimulation was applied using an Agilent 8110A pulse generator (Hewlett-Packard Co., USA). Furthermore, the optical transmittance of the chitosan–SWCNT RS layer was measured from 190 to 1100 nm using an Agilent 8453 UV–visible spectrophotometer (Hewlett-Packard Co., USA). Optical microscopy images of the prepared flexible memristors were obtained at 150× magnification using a Sometech SV-55 video microscopy system (Seoul, Korea). Figure 2a shows the optical transmittance spectra of the chitosan nanocomposite memristors with and without the SWCNT random network. The inset shows the expanded view between transmittance values of 88% and 92% for visible wavelengths (380–800 nm). Figure 2b shows an optical microscope image of the chitosan–SWCNT nanocomposite memristors.

## 3. Results and Discussion

**Figure 3:**
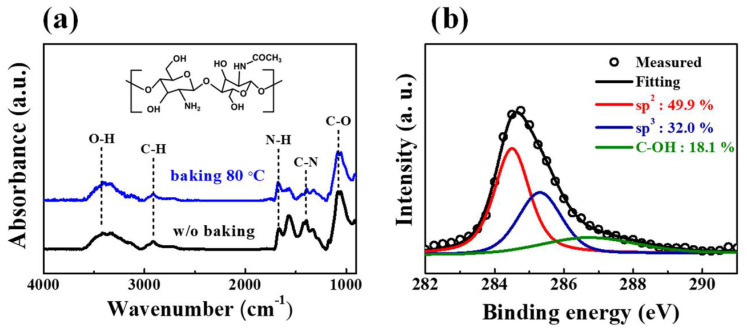
(**a**) FT-IR spectra of the chitosan electrolyte. Inset is the molecular structure of the chitosan electrolyte. (**b**) XPS C1s peak of the single-walled carbon nanotubes (SWCNTs).

Figure 3a shows the Fourier-transform infrared spectroscopy (FT-IR) spectra of the chitosan electrolyte with and without baking at 80 °C. After baking, the representative chitosan FT-IR peaks were observed. The O–H and C–H stretching peaks were observed around 3412 and 2912 cm^−1^, respectively. In addition, we observed N–H bending of –NH_2_ at 1672 cm^−1^, the C–N (amide) peak at 1398 cm^−1^, and the C–O peak at 1066 cm^−1^. Synaptic transistor experiments with a chitosan electrolyte as the EDL usually report these bands [28,29]. The inset in Figure 3a shows the molecular structure of the chitosan electrolyte. Figure 3b shows X-ray photoelectron spectroscopy (XPS) spectra, specifically the C1s peak, of the SWCNT random network. The spectra were measured after etching the surfaces of the CNTs with Ar^+^ ions to a depth of a few nanometers to validate the chemical composition of the CNTs. According to the chemical bonding state, we deconvoluted the C1s peak into three peaks. The sp^2^ peak (284.5 eV) is the primary peak of the graphite structure, the sp^3^ peak (285.3 eV) is related to irregular carbon atoms, and the C–OH peak (286.7 eV) is attributed to contaminants [30,31].

**Figure 4:**
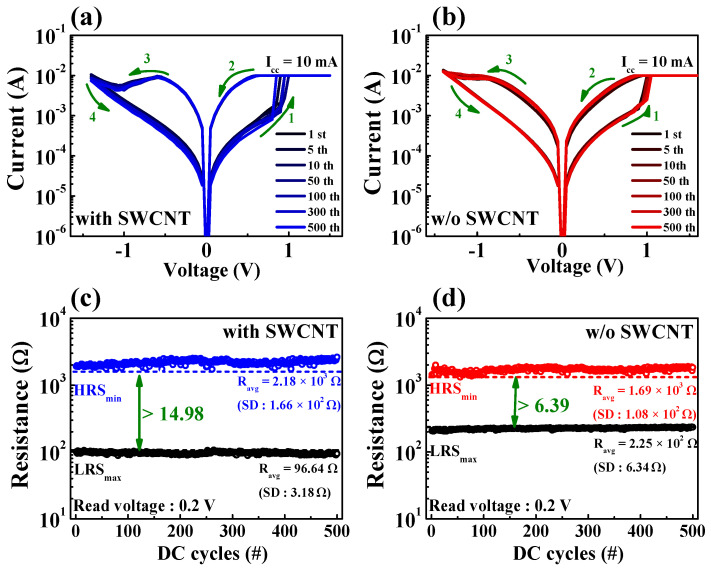
Results of resistive switching endurance tests over 500 DC cycles. BRS *I*-*V* curves of chitosan nanocomposite memristors (**a**) with and (**b**) without SWCNT random networks. Resistance states of the chitosan nanocomposite memristors (**c**) with and (**d**) without SWCNT random networks.

Figure 4a,b shows the endurance characteristics (over 500 DC cycles) of the nanocomposite memristors prepared with and without SWCNTs, which were investigated by applying a DC bias to the TE while the BE was grounded. When a positive voltage is applied to the TE (as indicated by green arrow 1), the memristor changes to the SET (ON) state, which corresponds to the resistance state changing from the high-resistance state (HRS) to the low-resistance state (LRS). Conversely, when a negative voltage is applied to the TE (as indicated by green arrow 3), the resistance state changes from LRS to HRS, resulting in a RESET (OFF) state. Consequently, both nanocomposite memristors showed bipolar RS (BRS) characteristics. Because of the redox reaction of mobile ions in the polymer electrolyte, the contact between the chitosan electrolyte and the electrode may be exploited for cation-based electrochemical conversion [11,32]. Electrochemical metallization reactions greatly influence the RS operation when an electric field is applied to an electrode of the chitosan nanocomposite memristors. Mobile cations are provided by chemically reactive metal electrodes, and their discharge causes the development of highly conductive filaments [33]. Figure 4c,d shows the resistance state extracted at a read voltage (*V*_read_) of 0.2 V for memristors with and without an SWCNT random network. The average resistances (*R*_avg_) of the HRS and LRS for memristors with SWCNT random networks were 2.18 × 10^3^ Ω and 96.64 Ω, respectively, with corresponding standard deviations (SDs) of 1.66 × 10^2^ Ω and 3.18 Ω. In contrast, for the HRS and LRS of memristors without SWCNT random networks, the *R*_avg_ values were 1.69 × 10^3^ Ω and 2.25 × 10^2^ Ω, respectively, with corresponding SDs of 1.08 × 10^2^ Ω and 6.34 Ω. Consequently, the RS memory window, which is defined as the minimum HRS (HRS_min_)/maximum LRS (LRS_max_), and the uniform resistance distribution of the nanocomposite memristors with SWCNT random networks were 2.3 times that of devices without SWCNTs. This is because the embedded SWCNT random network affects the interface dynamics of the chitosan thin-film layer via the adsorption of metal ions [34], which affects its interface dynamics, resulting in a large memory window through stable multilevel RS operation [35].

**Figure 5:**
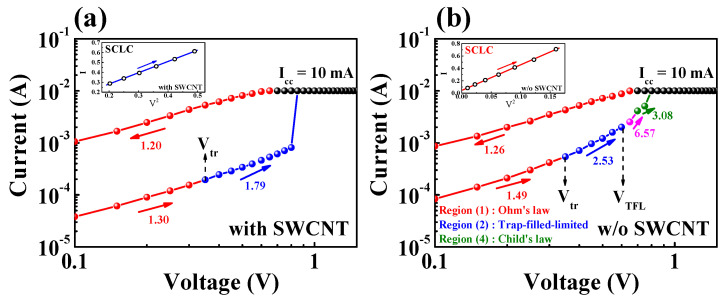
SCLC mechanism shown via double-logarithmic *I–V* curves of the chitosan nanocomposite memristors (**a**) with and (**b**) without SWCNTs. The HRS *I–V* curves at a high voltage are shown in the insets, which are well fitted by the SCLC mechanism.

To characterize the RS properties of chitosan nanocomposite memristors, the current mechanism was analyzed from current–voltage (*I*–*V*) curves of the SET operating region. Figure 5 shows *I*–*V* curves on a double-logarithmic scale for the chitosan nanocomposite memristors in the (a) presence or (b) absence of SWCNTs, showing the relationship of space-charge-limited conduction (SCLC) mechanisms. In the SET operation region, the chitosan nanocomposite memristor with an SWCNT random network showed two distinct sections, while the memristor without SWCNTs showed four sections. In region 1, the memristors follow the Ohmic law because the electric field applied to the RS layer is low and the number of thermally generated free charge carriers is greater than the few carriers injected into the RS layer [12]. As the voltage increases, the current rises nonlinearly and the slope of the curve changes in three sections as a result: trap filled limited (TFL) in region 2, a rapid increase in current in region 3, and Child’s law in region 4 [36]. In region 2, the memristor device is converted to TFL operation from the transition voltage (*V*_tr_). The number of injected carriers is greater than that of the thermally generated carriers in this situation, resulting in a steeper slope of the curve than that in region 1. The injected carriers are trapped in the shallow trap of the RS layer, and the mechanism is limited by the *I*–*V*^2^ relationship. The slope of the current curve steepens when the shallow traps are filled with carriers, correlating to the TFL voltage (*V*_TFL_). In region 4, all trapped carriers behave similar to space charges, which follow Child’s law, and the slope of the curve follows the *I*–*V*^2^ relationship [36,37,38]. The space charges are attributed to electrons injected from the electrode, dopant ionization at the interfacial depletion region, or the accumulation of mobile ions at the electrode interface [39,40,41,42]. The fitted *I–V* curves in the high-voltage section are shown as insets in Figure 5, and they match well the *I* ∝ *V*^2^ relationship. The *I*–*V* characteristics in the 1.5 → 0 V region follow a linear relationship after the SET operation, suggesting the development of a filament conduction path that is maintained until the RESET operation. Equations (1–3) were used to express the current density of each region.

(1) $$J_{Ohm}=qn_{0}\mu \frac{V}{d}$$

(2) $$J_{TFL}=\frac{9}{8}\mu \varepsilon \theta \frac{V^{2}}{d^{3}}$$

(3) $$J_{Child}=\frac{9}{8}\mu \varepsilon \frac{V^{2}}{d^{3}}$$

**Figure 6:**
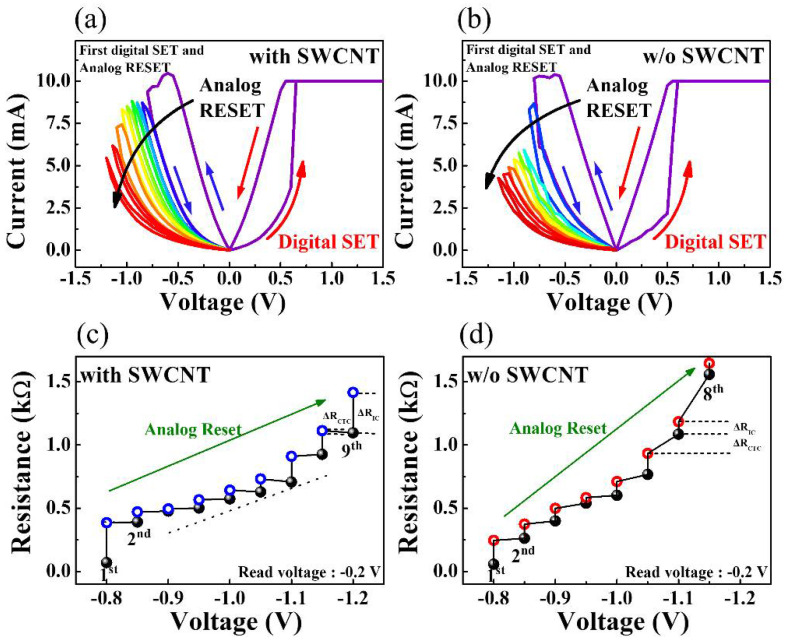
Analog RESET process of chitosan memristors (**a**) with and (**b**) without SWCNT random networks. Changes in resistance extracted at a read voltage of −0.2 V for chitosan memristors (**c**) with and (**d**) without SWCNT random networks.

Figure 6a,b shows the analog RESET process for chitosan nanocomposite memristors with and without SWCNT random networks. The analog RESET characteristics were measured by consecutively decreasing the maximum negative RESET voltage in steps of −0.05 V after performing one positive digital SET operation by applying a DC bias loop of 0 V → 1.5 V → 0 V. Figure 6c,d shows the change in resistance at *V*_read_ = −0.2 V in Figure 6a,b. The closed symbols show the resistance values when the TE bias decreases from 0 V to the maximum RESET voltage (*V*_reset−max_) in a RESET cycle, while the open symbols show the resistance when the TE bias increases from *V*_reset−max_ to 0 V. Therefore, the difference in resistance between the two symbols at the same *V*_reset−max_ (∆*R*_IC_, i.e., the in-cycle resistance change) represents the increase in the current according to *V*_reset−max_, and the change in resistance between the two symbols between *V*_reset−max_ values (∆*R*_CTC_, i.e., the cycle-to-cycle resistance change) represents the current retention over time. The results indicate that a consecutive increase in the resistance, a common analog property, stems from an increase in the *V*_reset−max_ value. The influence of the embedded SWCNT random network is evident in the ∆*R*_CTC_ value, which reveals that the resistance is maintained until a new stimulus is applied. For the chitosan nanocomposite memristor with an SWCNT random network, ∆*R*_CTC_ was constant, whereas it increased in the device without an SWCNT random network. This confirms that chitosan nanocomposite memristors with SWCNT random networks have less volatile behavior than those without. Therefore, the chitosan–SWCNT nanocomposite device is expected to provide reliable memristive switching.

**Figure 7:**
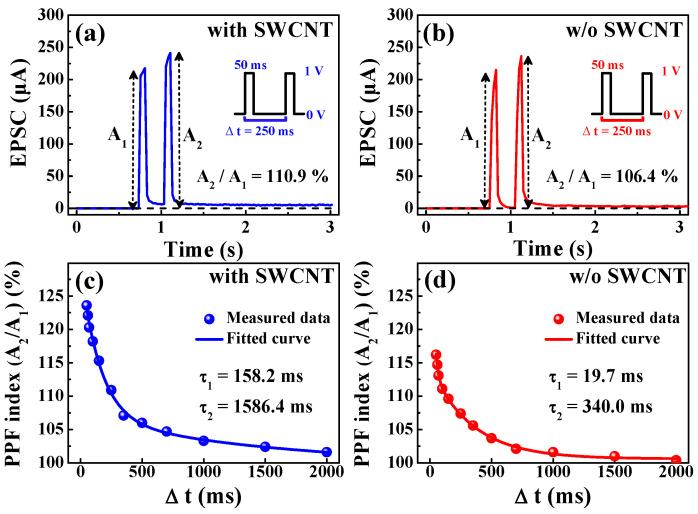
Triggered EPSC by PPF (amplitude: 1 V; duration: 50 ms; interval time: 250 ms) of chitosan memristors (**a**) with and (**b**) without SWCNT random networks. PPF indexes for chitosan memristors (**c**) with and (**d**) without SWCNT random networks.

Paired-pulse facilitation (PPF) is a neural facilitation phenomenon in which the post-synaptic potentials stimulated by an impulse are increased when that impulse closely follows the previous one and is an excitatory response between contiguous synaptic connections, which is an important property of short-term synaptic plasticity. The mobile protons transported by the first pre-synaptic spike accumulate between the electrolyte and the interface. If the interval time (∆*t*) is short, there is insufficient time for the mobile protons to return to their original positions, resulting in the continuous accumulation of mobile protons at the interface [43]. Figure 7a,b shows the excitatory post-synaptic currents (EPSCs) triggered by paired pre-synaptic spikes (amplitude: 1 V; duration: 50 ms; ∆*t* = 250 ms) for chitosan nanocomposite memristors with and without SWCNT random networks. In both memristors, the second EPSC peak is larger than the first (*A*_2_ > *A*_1_). This PPF property is attributed to partially relaxed mobile protons that are driven by paired pre-synaptic spikes as a function of ∆*t*. Figure 7c,d shows the PPF index as a function of ∆*t* for two consecutive pre-synaptic pulses, which is determined by a combination of the ratio of the EPSC peak amplitudes (*A*_2_/*A*_1_) and ∆*t*. In both devices, when ∆*t* is short, the PPF index increases, but when ∆*t* is long, it decreases, mimicking a biological synaptic response [44]. The measured PPF index data were fitted with the following double exponential decay relation [45]:

(4) $$PPF\;index=A+C_{1}exp\left(-\frac{\Delta t}{\tau_{1}}\right)+C_{2}exp\left(-\frac{\Delta t}{\tau_{2}}\right),$$

where *A* is a constant, *C*_1_ and *C*_2_ are the original facilitation magnitudes, and *τ*_1_ and *τ*_2_ are characteristic relaxation times. As indicated by the solid lines, the double exponential decay relation provides a close approximation of the PPF exponential-decay process. In this neural synapse model, *τ*_1_ is 436 ms and *τ*_2_ is 532 ms, which is consistent with the results obtained in biological synapses [46], indicating that our chitosan nanocomposite memristors can effectively emulate the PPF process of biological synapses. For the artificial synapse with an SWCNT random network, *τ*_1_ and *τ*_2_ were 158.2 and 1586.4 ms, respectively, whereas in the artificial synapse without an SWCNT random network, *τ*_1_ and *τ*_2_ were 19.7 and 340 ms, respectively. These fitting results are consistent with typical biological synapses, indicating that the proposed device can subdivide synaptic timescales into fast and slow steps lasting tens to hundreds of milliseconds [47].

**Figure 8:**
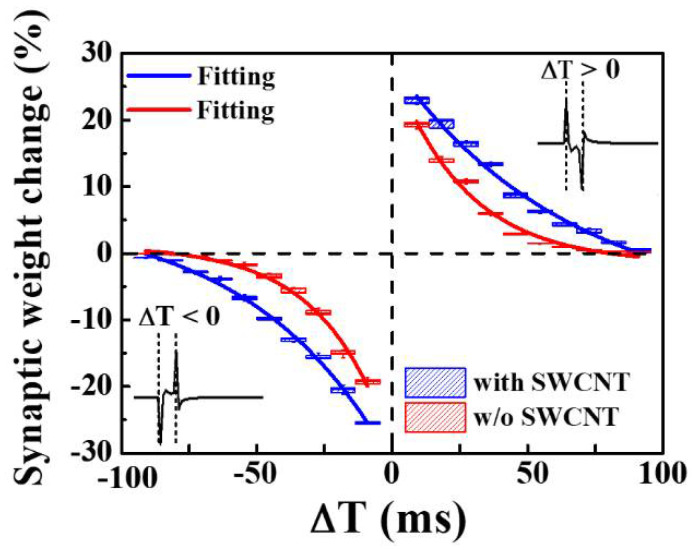
The STDP for the excitatory response mode of chitosan nanocomposite memristors with and without SWCNT random networks. The inset show illustrations of the spike signals.

Figure 8 shows the spike-timing-dependent plasticity (STDP) characteristics for the excitatory response mode. Note that STDP is the ability of natural or artificial synapses to change their strength depending on the precise timing of individual pre- and/or post-synaptic spikes (*I*_1_ and *I*_2_, respectively). The synaptic weight changes as a function of the relative timing (∆*T* = *t*_post_ − *t*_pre_) between the arrival time of the pre-synaptic spike (*t*_pre_) and the post-synaptic spike generation time (*t*_post_). When the pre-synaptic spike appears before the post-synaptic spike (∆*T* > 0), the synaptic weight increases, strengthening the synaptic connection (i.e., potentiation). In addition, the smaller the spike timing difference, the greater the change in the synaptic weight. Conversely, when the post-spike precedes the pre-spike (∆*T* < 0), the synaptic weight decreases, meaning that the synaptic connection is inhibited (i.e., depression). The change in the synaptic weight decreases as ∆*T* increases. A pair of pulses (+1 V, 10 ms and −1 V, 10 ms) was implemented as the pre- and post-synaptic spikes to emulate the STDP rule. When the STDP was inverted, the same device was switched to the inhibitory response mode [43]. The relative synaptic weight change is defined as ∆*W* = (*I*_2_ − *I*_1_), yielding a positive value for ∆*T* > 0 and a negative value for ∆*T* < 0. The fitting lines describing the STDP behavior in each response mode are:

(5) $$\Delta W=\{\begin{array}{c} A^{+}exp\left(-\Delta T/\tau^{+}\right),\;\;\;\;\;\;\;\Delta T\geq 0\\ -A^{-}exp\left(\Delta T/\tau^{-}\right),\;\;\;\;\;\;\;\;\;\Delta T<0 \end{array}.$$

The range of ∆*T* is given by *τ*^+^ and *τ*^−^, which represent the ranges in which the potentiation and depression of synaptic connections occur, respectively. In addition, *A*^+^ and *A*^−^ determine the maximum amount of synaptic modification that occurs when ∆*t* is close to zero [48,49,50]. As a result, the synaptic weight change (in percent) of memristors with SWCNT random networks is larger than that of memristors without SWCNT random networks. The precise pre- and post-spike timing windows that control the sign and magnitude of synaptic weight modifications are approximately 100 ms for biological synapses [51,52]. The reinforcement and inhibition of synaptic connections between two neurons are known as long-term potentiation (LTP) and long-term depression (LTD), respectively [53]. Because of its simplicity, biological relevance, and computational power, the STDP is the subject of significant attention in neuroscience.

**Figure 9:**
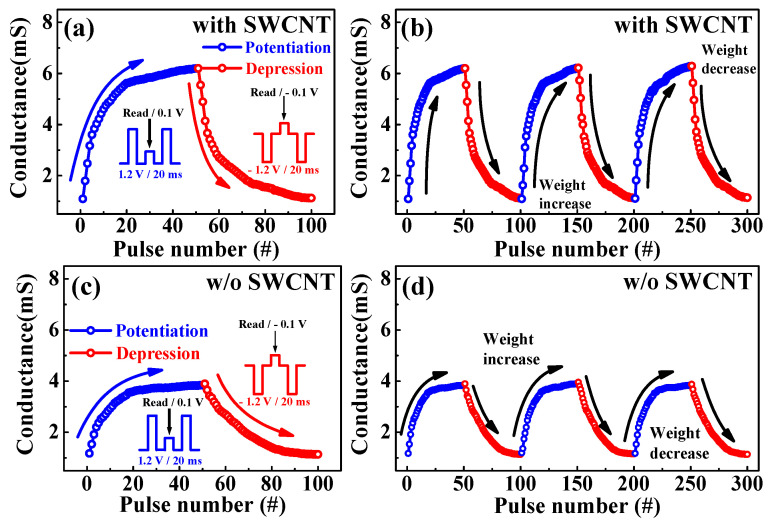
Potentiation and depression behavior of chitosan nanocomposite memristors (**a**) with and (**c**) without SWCNT random networks. Cycle testing under stimulation from 300 synaptic pulses for memristors (**b**) with and (**d**) without SWCNT random networks.

Figure 9 shows the analog synaptic weight change characteristics in the chitosan nanocomposite memristors with and without embedded SWCNT random networks, thus revealing the change in the conductance through repeated reinforcement (weight increase) and degradation (weight decrease). Figure 9a,c shows the conductance change characteristics in one cycle consisting of 50 potentiation pulses and 50 depression pulses. The pulse conditions for potentiation and depression were +1.2 V for 20 ms and −1.2 V for 20 ms, respectively. As shown in Figure 9a, the dynamic range of conductance modulation was ~5.1 mS for the memristor with an SWCNT random network, which decreased to ~2.6 mS for the memristor without an SWCNT random network (Figure 9c). Figure 9b,d shows the successive conductance increase and decrease behaviors over 300 synaptic pulses, which provides insights into the reliability of the weight modulation. The results show that during cycle repetition, memristors with SWCNT random networks operate stably with a large dynamic range of conductance, whereas memristors without SWCNT random networks show small conductance fluctuations. Thus, the embedded SWCNT random network is effective for improving the uniform weight modulation and memory behavior of memristors in response to stimulation from potentiation/depression pulses, indicating its applicability for artificial synaptic devices. In addition, it is expected that the efficiency of the learning process will be amplified because the chitosan nanocomposite memristors with embedded SWCNT random networks have higher conductance than those without CNTs [54,55].

## 4. Conclusions

High-performance two-terminal memristors were fabricated on flexible, transparent PEN substrates using RS layers of SWCNT random networks embedded in chitosan layers. The resistive and memristive switching characteristics, STDP, and learning process of the devices were evaluated. To pinpoint the influence of the SWCNT random network, memristors were prepared with and without SWCNT random networks using low-temperature solution processing. Both devices were transparent at visible wavelengths (380–800 nm) and demonstrated BRS behavior; however, a larger memory window was identified in the devices with SWCNT random networks, where metal-ion adsorption leads to the formation of highly conductive filaments at the interface with the chitosan layer. In addition, the chitosan memristors with SWCNT random networks had improved analog synaptic properties regarding their memristive switching behavior, which more closely emulated the long- and short-term plasticity associated with PPF and STDP, respectively, because the synaptic weight change was larger than for memristors without SWCNTs. As such, the flexible, transparent chitosan–SWCNT memristor devices proposed here show great promise as artificial synaptic devices for future applications such as solar cells, supercapacitors, and EDLCs.
